# P-1379. Congenital Syphilis in Southeast Michigan: A Retrospective Case Series

**DOI:** 10.1093/ofid/ofae631.1555

**Published:** 2025-01-29

**Authors:** Diana D D Cardenas-Maldonado, Smitha Gudipati, Maria Santana-Garcés, Gina Maki, Indira Brar

**Affiliations:** Henry Ford Hospital, Farmington Hills, Michigan; Henry Ford Hospital, Farmington Hills, Michigan; Henry Ford Health, Livonia, Michigan; Henry Ford Health System, Detroit, MI; Henry Ford Hospital, Farmington Hills, Michigan

## Abstract

**Background:**

Cases of congenital syphilis (CS) have been increasing substantially, but CS is preventable through timely testing and adequate treatment of syphilis during pregnancy. From 2012 to 2021, cases of CS in the United States increased by 755%. Our study aims to describe the demographics of pregnant persons infected with syphilis and outcomes in their infants to identify potential predictors of adverse events.

**Figure:**
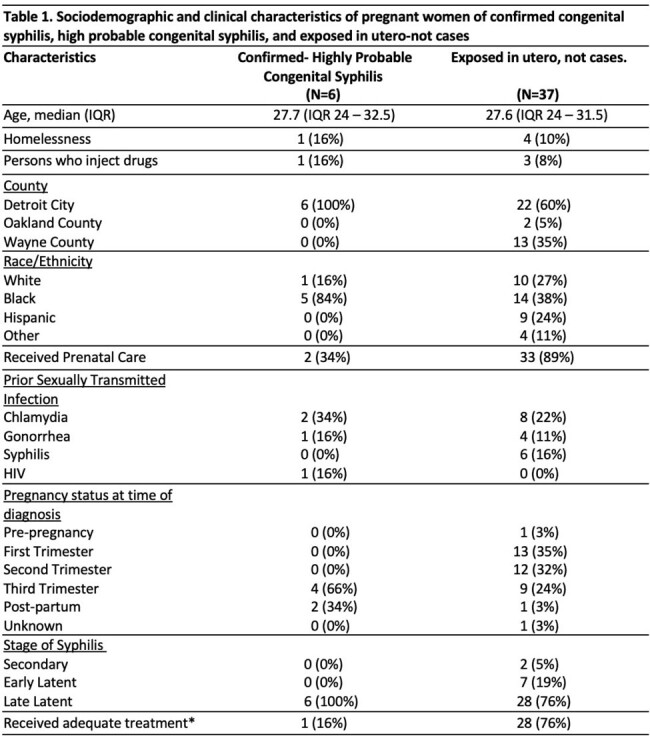

* Received adequate treatment: Complete Penicillin treatment in pregnant patients, thirty days before delivery

IQR: Interquartile range; HIV: human immunodeficiency virus;

**Methods:**

This is a retrospective case series of pregnant persons diagnosed with syphilis who delivered their babies at Henry Ford Health from January 2020 to January 2024. Charts were reviewed for demographic data, syphilis serology, treatment in mothers and infants, and infant outcomes. Descriptive statistics were used to report the study population’s demographic and clinical characteristics. Chi-squared tests were used to determine the association between receipt of penicillin, prenatal care, prior sexually transmitted infection (STI), and CS diagnosis in neonates. Our chosen significance level was 0.05.

**Figure:**
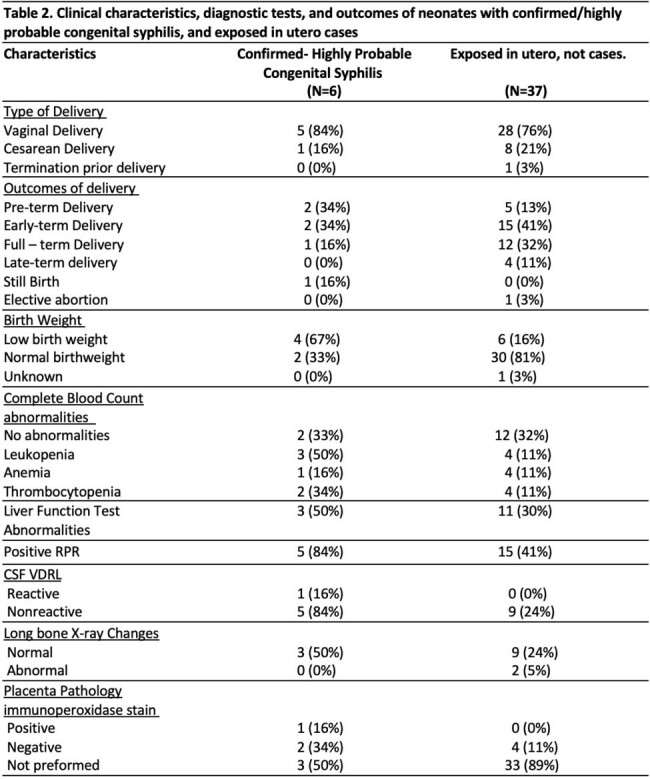

RPR: rapid plasma reagin; CSF: Cerebrospinal fluid; VDRL: Venereal disease research laboratory test.

**Results:**

Forty-three patients were included; 19 (44%) were black; median age 27 years (IQR 24-32.5); 28 (66%) were from Detroit; 18 (53%) reported a history of STI. Eight (18%) did not receive prenatal care. Syphilis was primarily diagnosed during second trimester (34%), and late latent syphilis was reported in 80%. Syphilis treatment was completed 30 days before delivery in 67%. There were 17 early-term deliveries, 1 stillbirth, and 1 elective abortion. Ninety percent of neonates had a normal physical exam. Fifteen lumbar punctures were performed, one positive Venereal Disease Research Laboratory test. Of the 8 placenta studies, 2 had positive immunoperoxidase treponemal stain. Seventeen neonates were treated with 10 days of aqueous Penicillin, 6 neonates had definite/highly probable CS. Using Chi-square analysis, receipt of penicillin treatment by the mother less than 30 days before delivery was significantly associated with CS in the neonate (p < 0.001)

**Conclusion:**

In our analysis, we identified a significant association between CS and appropriate timing of treatment of syphilis in the mother. Further studies are needed with a larger cohort to determine if social determinants of health affect appropriate prenatal care in pregnant persons.

**Disclosures:**

**Indira Brar, MD**, Gilead: Advisor/Consultant|Gilead: Grant/Research Support|Gilead: Honoraria|Merck: Grant/Research Support|ViiV Healthcare: Grant/Research Support|ViiV Healthcare: Honoraria

